# Non-Canonical, Extralysosomal Activities of Lysosomal Peptidases in Physiological and Pathological Conditions: New Clinical Opportunities for Cancer Therapy

**DOI:** 10.3390/cells14020068

**Published:** 2025-01-07

**Authors:** Ryan Conesa-Bakkali, Macarena Morillo-Huesca, Jonathan Martínez-Fábregas

**Affiliations:** 1 Centro Andaluz de Biología Molecular y Medicina Regenerativa—CABIMER, Universidad de Sevilla, Consejo Superior de Investigaciones Científicas (CSIC), Universidad Pablo de Olavide, Américo Vespucio 24, 41092 Sevilla, Spain; ryan.conesa@cabimer.es (R.C.-B.); macarena.morillo@cabimer.es (M.M.-H.); 2 Departamento de Bioquímica Vegetal y Biología Molecular, Facultad de Biología, Universidad de Sevilla, Avenida Reina Mercedes, 41012 Sevilla, Spain

**Keywords:** lysosomes, proteases, nucleus, cytosol, extralysosomal activities, cancer

## Abstract

Lysosomes are subcellular compartments characterised by an acidic pH, containing an ample variety of acid hydrolases involved in the recycling of biopolymers. Among these hydrolases, lysosomal proteases have merely been considered as end-destination proteases responsible for the digestion of waste proteins, trafficked to the lysosomal compartment through autophagy and endocytosis. However, recent reports have started to unravel specific roles for these proteases in the regulation of initially unexpected biological processes, both under physiological and pathological conditions. Furthermore, some lysosomal proteases are no longer restricted to the lysosomal compartment, as more novel non-canonical, extralysosomal targets are being identified. Currently, lysosomal proteases are accepted to play key functions in the extracellular milieu, attached to the plasma membrane and even in the cytosolic and nuclear compartments of the cell. Under physiological conditions, lysosomal proteases, through non-canonical, extralysosomal activities, have been linked to cell differentiation, regulation of gene expression, and cell division. Under pathological conditions, these proteases have been linked to cancer, mostly through their extralysosomal activities in the cytosol and nuclei of cells. In this review, we aim to provide a comprehensive summary of our current knowledge about the extralysosomal, non-canonical functions of lysosomal proteases, both under physiological and pathological conditions, with a particular interest in cancer, that could potentially offer new opportunities for clinical intervention.

## 1. Introduction

**Figure 1:**
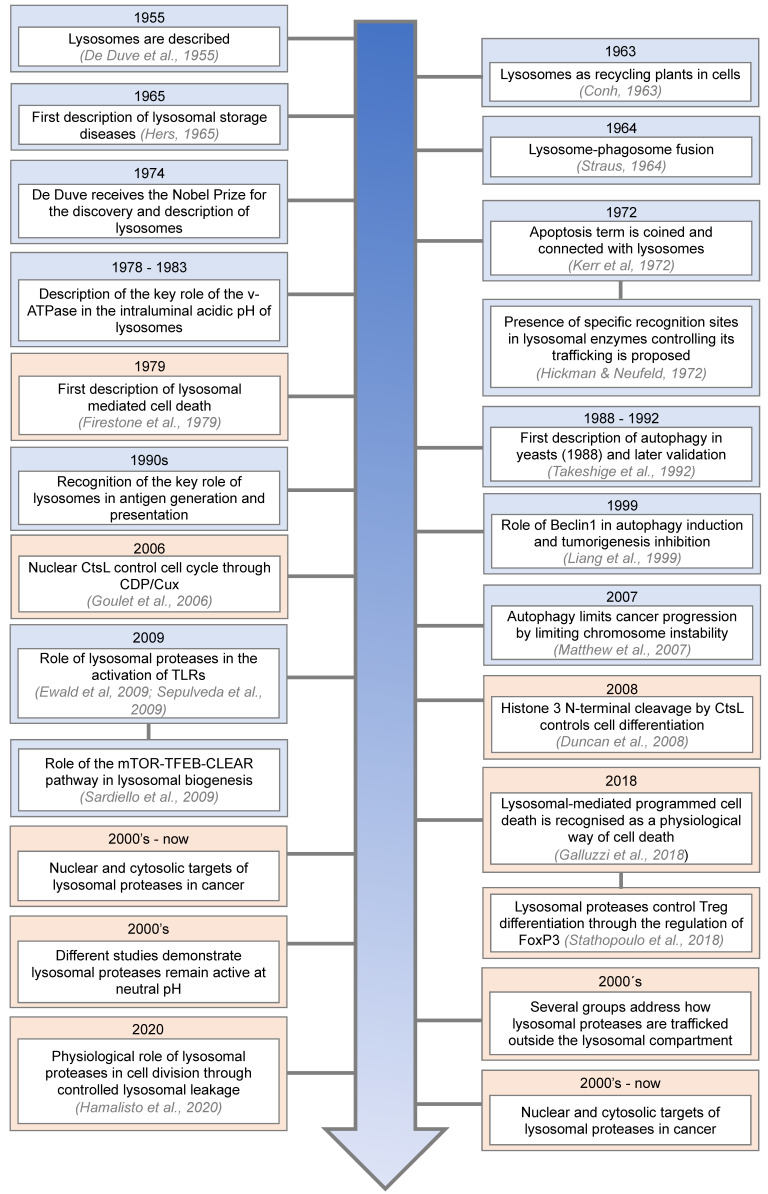
Timeline showing the main discoveries in lysosomal biology. In red boxes, some of the non-canonical, extralysosomal functions of lysosomal proteases are identified. In blue boxes, some of the main milestones in lysosome research are presented [4,5,6,7,8,9,10,11,12,13,14,15,16,17,18,19,20,21].

Lysosomes, subcellular organelles found in most eukaryotic cells, are responsible for the degradation and recycling of extracellular material, previously internalised by endocytosis [1] and/or phagocytosis [2], and intracellular components sequestered by autophagy [3]. Since their discovery by Christian de Duve in 1955 [4], lysosomes have been the subject of numerous important studies and discoveries, allowing for a better understanding of their role in the cell (Figure 1). These studies have allowed us to change our view of lysosomes from simple recycling centres to complex signalling hubs involved in the regulation of multiple physiological processes.

Lysosomes are membrane-limited, subcellular components characterised by an acidic intraluminal pH, filled with a plethora of acid hydrolytic enzymes, including lipases, nucleases, glycosidases, sulphatases, proteases, etc, that are fundamental for the role of lysosomes in the degradation and recycling of biopolymers. In this regard, lysosomes are packed with more than fifty different acid hydrolases, with lysosomal proteases representing the largest group [22], that allow lysosomes to play their role as the recycling plant of the cell. These proteases can be organised into three well-defined, structurally unrelated families: pepsin-like aspartyl cathepsins (cathepsins (Cts) D and CtsE), papain-like cysteine cathepsins (CtsB, CtsC, CtsF, CtsH, CtsK, CtsL, CtsO, CtsS, CtsV, CtsW, and CtsX/Z), chymotrypsin-like serine cathepsins (CtsA and CtsG) and finally, a structurally unrelated cysteine protease known as legumain or asparagine endopeptidase (AEP), which is closely related to caspases and separase [23,24,25]. However, lysosomes are nowadays recognised as much more than mere recycling centres, being key players in the regulation of the immune system [15,16,26,27,28,29] and the normal physiology of the cell [24,30,31,32]. This renewed perception of lysosomes has allowed us to rationalise their key role in the onset and progression of a plethora of human diseases, such as lysosomal storage diseases (LSDs) [33], neurodegenerative diseases [34], autoimmune disorders [35], and cancer [36,37]. Importantly, until quite recently, these processes were thought to rely on proteolytic activities constrained to the lysosomal lumen due to earlier works reporting their in vitro denaturation at neutral pH [38]. These functions taking place within the lysosomal compartment constitute the so-called canonical, intralysosomal functions of lysosomal proteases. However, as highlighted in Figure 1, recent, growing evidence has demonstrated, at least in some instances, that lysosomal proteases remain active at neutral pH, albeit in some cases with modified enzyme kinetics and substrate specificity [39,40,41,42]. Moreover, a growing body of literature has confirmed their nuclear and cytosolic localisation, demonstrating that they remain active in these extralysosomal locations, where they play critical roles in initially unexpected processes, both under physiological and pathological conditions (Figure 1). In this regard, lysosomal proteases, through their extralysosomal activity—so-called non-canonical activities— have been linked to cell division [21,43,44], programmed cell death [43,44], neurotoxicity [45,46,47], immune cell differentiation [20,27], gene expression [17] and cancer [48,49,50,51]. However, one of the most exciting questions in the field remains to be addressed: How are lysosomal proteases trafficked to the cytosol or nuclear compartments of the cell? In this context, some seminal works, which will be discussed later, have started to unveil the molecular mechanisms controlling this key event [21,52,53,54] (Figure 1). Interestingly, non-canonical, extralysosomal functions have also been reported for other lysosomal hydrolases, such as glucosylceramidase beta 1 (Gba1), further reinforcing the non-canonical, extralysosomal role of these hydrolases, including lysosomal proteases, in the regulation of biological processes, both under physiological and pathological conditions [55,56,57].

The identification of the biologically relevant targets of some of these lysosomal proteases, confirming their role in the regulation of initially unexpected biological processes, both under physiological and pathological conditions, offers new therapeutical opportunities for the treatment of a plethora human diseases. In this context, this manuscript aims to provide an updated, comprehensive review of the non-canonical, extralysosomal biological targets and processes regulated by these proteases, both under physiological and pathological conditions, with special emphasis in cancer.

## 2. Canonical, Intralysosomal Functions of Lysosomal Proteases and Disease

**Figure 2:**
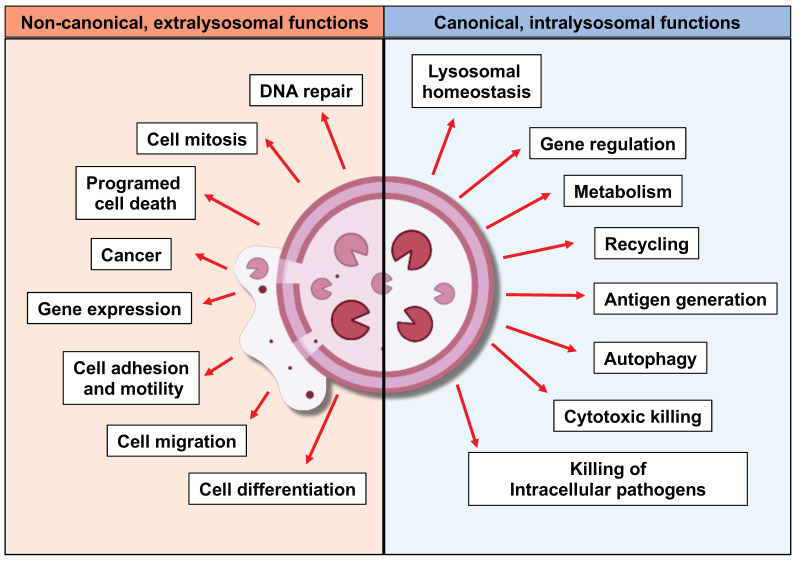
Scheme showing the main canonical, intralysosomal (right, blue box) and non-canonical, extralysosomal (left, red box) functions described throughout this review.

Together, all these lysosomal proteases contribute to the function of lysosomes as recycling plants, responsible for the bulk degradation and turnover of waste and endogenous proteins [58]. Furthermore, within the lysosomal compartment, they are involved in a myriad of processes, including the clearance of internalised pathogens [59,60,61], pathogen detection and signalling [15,16,62], processing and presentation of endogenous and foreign antigens [61,63], activation of chemokines and cytokines [62,64], regulation of cell signalling through the proteolytic degradation of cell surface receptors [65,66], regulation of lysosomal homeostasis [24], and cellular metabolism [67,68] (Figure 2).

All these activities occurring within the lysosomal compartment constitute the canonical functions of lysosomal proteases. Importantly, dysregulation of these cellular processes is linked to the onset and progression of a wide variety of human diseases. Moreover, deletion of individual murine lysosomal proteases results in clear tissue-specific phenotypes/diseases, thus strengthening the idea that they have non-redundant functions [53,69,70,71]. In this context, the lack of specific lysosomal proteolytic activities has been associated to different forms of lysosomal storage diseases [72,73,74]; meanwhile, dysregulation of the lysosomal activity is directly linked to the appearance and progression of different diseases, such as autoimmune diseases and cancer [75,76,77,78,79,80,81].

### 2.1. Bulk Protein Degradation and Lysosomal Storage Diseases

As previously described, lysosomes are packed with a complete set of acid hydrolytic enzymes. Unneeded and damaged biomolecules (such as proteins, nucleic acids, carbohydrates, lipids, etc.) as well as damaged subcellular compartments are continuously targeted to the endolysosomal compartment for their degradation and recycling (Figure 2). In combination, all these hydrolases are required for the complete degradation of this cargo within the lysosomal compartment [82,83,84,85,86,87]. Remarkably, this lysosomal function is well conserved throughout evolution, thus highlighting its key role in the maintenance of cellular homeostasis [88,89,90,91].

In this context, the lack of specific lysosomal hydrolases drives the accumulation of specific, undigested molecules, hence leading to the onset and progression of different LSDs. LSDs represent a group of more than 70 different, rare inborn metabolic alterations, genetically unrelated, that are associated to mutations in proteins involved in the degradation or transport of macromolecules or in modulators of the lysosomal microenvironment [33,92]. Even though, individually, LSDs affect a low number of patients, as a group, their incidence increases up to 1:5000 [93,94,95], thus highlighting its clinical relevance. Some examples of these diseases are shown in Table 1, indicating the gene mutation associated to its onset.

In most cases, LSDs are linked to mutations in hydrolases and lysosomal transporters, rather than lysosomal proteases [33]. However, there are some cases in which the mutation of specific lysosomal proteases has been shown to drive the onset and progression of LSDs. Specifically, in mouse models, the loss of some lysosomal proteases (e.g., *CTSA* [72], *CTSB* [96], *CTSD* [97,98], *CTSF* [74], *CTSK* [99], *CTSL* [96] and *CTSS* [97]) results in the onset and progression of LSDs, while in humans, mutations in some lysosomal proteases have been linked to LSDs.

In this regard, specific mutations in some lysosomal proteases in human patients have been linked to the onset and progression of different forms of lipidoses. In humans, several pathogenic mutations in the gene encoding CtsD are linked to the congenital, late infantile or juvenile onset of type 10 Neuronal Ceroid Lipofuscinosis (NCL) [100,101,102,103,104,105,106], a severe neurodegenerative LSD characterised by the accumulation of autofluorescent lipopigments [107]. These mutations trigger different degrees of neuropathogenesis, depending on the degree of CtsD inactivation (reviewed in detail in [108]). Similarly, several mutations in the *CTSF* gene leading to the onset of type 13 NCL have been identified in humans [74,109,110,111]. Moreover, despite the lack of identified mutations in *CTSB* and *CTSL* leading to LSDs in human patients, mouse models with both *CTSB* and *CTSL* deficiencies and the double knock-out develop a neuropathology that resembles human NCL [112]. Furthermore, a deficiency in *CTSB* and *CTSL* in human neuroblastoma cells results in the accumulation of cholesterol in late endosomes/lysosomes, leading to a neuropathology that resembles Niemann–Pick disease type C, also included among lipidoses [96] (Table 1).

Pycnodysostosis is a rare, autosomal recessive LSD characterised by the abnormal hardening of the bones and reduced stature [99] (Table 1). With respect to this, nonsense, missense, and stop codon mutations in the *CTSK* gene, leading to CtsK deficiency, have been identified in human patients.

Finally, in humans, *CTSA* mutations leading to loss of or reduced CtsA activity are linked to the onset of Galactosialidosis, also known as neuraminidase deficiency with β-galactosidase deficiency, which is included among glycoproteinoses [72] (Table 1). In normal conditions, CtsA forms a complex with beta-galactosidase (*GLB1*) and neuraminidase 1 (*NEU1*) to properly degrade glycoproteins [113]. However, mutations in the *CTSA* gene, affecting its interaction with GLB1 and NEU1, or its deficiency, lead to the destabilisation and degradation of these enzymes, resulting in the accumulation of undigested material and the onset of this LSD.

### 2.2. Innate and Adaptive Immunity and Autoimmune Diseases

Lysosomal activity directly influences the regulation of the innate and adaptive immune responses [63,114]. In this context, lysosomes play a key role in the detection and signalling of pathogens through the activation of Toll-like receptors (TLRs) [115]. Furthermore, lysosomes are essential in the processing and presentation of antigens derived from both pathogens and endogenous proteins [116] (Figure 2). For this reason, lysosomes, and more specifically lysosomal proteases, through their canonical, intralysosomal activity, are essential for the activation of proper immune responses, but also for the acquisition of self-tolerance. Therefore, it should not come as a surprise that lysosomal dysfunction has been linked to the onset and progression of a plethora of autoimmune diseases [35,64,117,118].

#### 2.2.1. Innate Immune Response

During the innate immune response, pathogens can be internalised through phagocytosis and targeted to the lysosomes to be digested by the lysosomal proteases. Furthermore, in the case of cytosolic pathogens and those that manage to escape the endolysosomal system gaining access to the cytosol, they can be captured through autophagy and targeted to the lysosomal compartment to be eliminated. In this context, the lysosomal compartment, and more specifically, the lysosomal proteases, serve as an intracellular defence system, eliminating pathogens and protecting the cells from infection [59] (Figure 2).

In addition, lysosomal proteases are responsible for the proteolytic processing necessary for the complete activation of TLRs, which are responsible for the detection of Pathogen-Associated Molecular Patterns (PAMPs), pathogen-specific molecules whose recognition by receptors of the innate immune system triggers a rapid and generalised immune response [119,120,121]. This response includes the production of pro-inflammatory cytokines, such as tumour necrosis factor (TNF) and interleukin-1 (IL-1), which promote inflammation and the recruitment of immune cells to the site of infection, and the induction of phagocytosis, a process by which phagocytic cells engulf and destroy invading microorganisms [15,16,115,117,118,119,122].

Finally, lysosomes are also involved in the innate immune response against virus-infected cells or cancer cells through secretory lysosomes. These specialised subcellular compartments are lysosome-related organelles characterised by the catabolic functions of lysosomes, while presenting inducible secretory capabilities [29,123,124]. Secretory lysosomes are present in both cytotoxic CD8^+^ T lymphocytes and Natural Killer (NK) cells, and they are packed with a full set of acid lysosomal hydrolases as well as lethal proteins such as perforins and granzymes [120,121] (Figure 2). When these cells encounter virus-infected host cells and cancer cells, the secretory lysosomes fuse with their plasma membrane, releasing their content to the extracellular milieu. Upon release, perforin forms a pore in the plasma membrane of the target cell, allowing granzymes and lysosomal proteases to trigger the activation of apoptosis [121,125,126].

#### 2.2.2. Adaptive Immune Response

The role of lysosomes in the regulation of the immune system also includes important functions in the adaptive immune response. In this regard, lysosomal proteases are essential players in the processing and presentation of antigens. In specialised antigen-presenting cells (APCs)—such as macrophages, dendritic cells, and B lymphocytes—both endogenous and pathogen-derived proteins are targeted to the lysosomes, where they are digested into smaller fragments to generate antigens. These antigens are subsequently loaded into Major Histocompatibility Complex II (MHC-II) molecules to be presented to CD4^+^ T cells. For these reasons, lysosomal proteases not only play an essential role in the activation of proper immune responses against specific pathogens, but also guarantee the recognition of self-antigens and the acquisition and development of self-tolerance [65,117,122,127] (Figure 2).

#### 2.2.3. Role of Lysosomal Proteases in Autoimmune Diseases

In autoimmune diseases, the immune system mistakenly attacks its own cells and tissues, leading to dysfunctions in various systems and organs. The role played by lysosomes in the central pathways of the immune system (including antigen processing and presentation, cytokine processing, activation of TLR-mediated signalling, etc.) justifies its contribution to the onset and progression of these diseases (Figure 2). Moreover, lysosomes play a multifaceted role in the onset and progression of these types of disorders, as increased levels of autophagy, high expression levels of specific lysosomal enzymes, and elevated luminal pH of cells have been confirmed in patients suffering from these types of disorders [64,66,84,128]. In this regard, the aforementioned alterations regarding the proper functioning of lysosomes can lead to the generation and presentation of new self-antigens, thus triggering aberrant immune responses against its own cells and tissues. Furthermore, lysosomes are also key in the activation of cytokines and chemokines in immune cells [61]; therefore, changes in the lysosomal compartment affecting this processing can affect the immune function of cells, thus leading to autoimmune diseases [61,129].

For all these reasons, lysosomes have been associated to the onset and progression of a wide variety of autoimmune diseases [123]. Interestingly, elevated expression levels of different lysosomal proteases have been linked to some of these pathologies, such as Systemic Lupus Erythematosus (SLE) [80,124,129,130,131], Rheumatoid Arthritis (RA) [127,128,132,133,134,135,136,137,138,139] and Amyotrophic Lateral Sclerosis (ALS) [127,128,132,133,134,135,136,137,138,139].

SLE is an autoimmune disorder characterised by the production of autoantibodies, aberrant inflammation, and multiple organ damage. The abnormal processing and presentation of antigens is considered one of the first events involved in the onset of the disease [140]. In this regard, increased levels of expression and activity of lysosomal proteases (CtsB, CtsD, CtsL, and CtsS, among others), known to portray critical roles in antigen processing and presentation, have been reported to be altered in SLE [83,134,136,137]. Furthermore, in a mouse model, CtsK was shown to be linked to SLE through the proteolytic processing and activation of TLR7 [131].

RA is an autoimmune disease in which the immune system wrongly attacks the joints, leading to inflammation, joint destruction, and bone damage [141]. In RA patients, lysosomes are overactive in inflammatory cells, showing increased levels of CtsB, CtsD, CtsG, CtsK, CtsL, and CtsS that contribute to most of the clinical manifestations of RA [127,128,132,133,134,135,136,137,138,139].

The causes of ALS remain vastly unknown; however, increasing evidence supports the presence of a dysregulated immune response contributing to the pathogenesis. With respect to this, increased expression levels of various lysosomal proteases, such as CtsB, CtsD, CtsX, and CtsZ, have been detected in patients with ALS and/or mouse models, thus revealing a potential role for these proteases in the onset and/or progression of this pathology [142,143,144,145,146,147], although the contribution of these increased levels to the onset and progression of ALS at the molecular level still remains to be determined.

### 2.3. Cancer

Beyond their classical role in protein turnover and antigen generation and presentation, lysosomes are known to play key functions in energy homeostasis, generation of building blocks for cell growth, and immune escape through their canonical, intralysosomal functions (reviewed in [36]) (Figure 2). In this context, lysosomal proteases, through their canonical, intralysosomal functions, have been shown to play central roles in the onset and progression of cancer.

Lysosomal proteases are responsible for the degradation of unneeded proteins within the lysosome, providing cells with building blocks for the synthesis of new proteins, thus promoting cell growth. Interestingly, cancer cells are characterised by accelerated rates of growth and increased demand for energy supply. This explains why cancer cells show increased lysosomal activity and autophagy, as they could allow them to sustain elevated proliferation rates in conditions when nutrients become limiting [148].

Moreover, lysosomes, through their central role in antigen generation and presentation and the activation of the immune response, can play a central role in immune escape in cancer through the degradation of specific antigens, thus justifying the increased lysosomal proteolytic activity observed in cancer cells [36,149].

## 3. Non-Canonical, Extralysosomal Roles of Lysosomal Proteases in Cancer

These canonical, intraluminal activities of lysosomal proteases have been considered, for a long time, as the only functions executed by these proteases. However, emerging evidence is revealing a non-canonical role for these proteases, highlighting their role in the regulation of key processes, both under physiological and pathological conditions, with a special relevance in cancer (Figure 2). Moreover, extracellular, cytosolic, and nuclear specific targets for some of these lysosomal proteases have been identified, thus revealing initially unexpected functions and increasing the number of biological processes regulated by these proteases (Figure 2). However, until recently, two main factors have limited the characterisation of these non-canonical, extralysosomal functions. First, the acidic lysosomal pH, considered essential for the activity of these lysosomal proteases, and reports indicating their in vitro denaturation at neutral pH have both limited the study of their extralysosomal activities [38]. Second, until recently, the lack of knowledge on the molecular mechanisms controlling the extralysosomal localisation of these proteases has further contributed to the idea that these non-canonical, extralysosomal activities were simple artifacts, with no relevant biological functions. In this review, we will summarise our current knowledge on 1. how lysosomal proteases maintain their activity at neutral pH and how changes in the pH affect their activity and specificity outside the lysosomal compartment, and 2. how they reach these subcellular compartments. Furthermore, we will present our current knowledge on the non-canonical, extralysosomal functions of these proteases under physiological and pathological conditions, with a special focus in cancer.

### 3.1. Effect of the pH in the Activity of Lysosomal Proteases

Lysosomes are characterised by an acidic pH around 4.5, in which lysosomal proteases remain folded, achieving their maximum activity, with some reports showing that lysosomal proteases become denatured and inactivated at neutral pH [38]. This idea has impacted the study and understanding of the non-canonical, extralysosomal activities of these proteases. However, recent reports have demonstrated that these proteases can be detected in the extracellular space, but also in the nuclear and cytosolic compartments of the cell, where they retain their activity.

In this regard, one of the first examples of these extralysosomal activities was described in 1992, when CtsB was shown to be involved in the degradation of the extracellular matrix (ECM), both under acidic and neutral pH [150]. However, later work demonstrated that a large array of lysosomal proteases (i.e., CtsF, CtsK, CtsL, CtsS and CtsV), and not just CtsB, are able to degrade the components of the ECM in the extracellular space, further demonstrating that they remain active at neutral pH [150,151,152] (Figure 2). Furthermore, some interactions have been shown to contribute to the stabilisation and activity of these proteases at neutral pH. In this context, interactions between CtsB and heparin, and high substrate concentrations in the case of CtsL, have been shown to contribute to the stabilisation of both proteases at neutral pH [39]. Furthermore, recent reports have confirmed that at least some of these proteases remain active outside the lysosomal compartment at neutral pH, albeit showing reduced enzyme kinetics and substrate specificity. Consistently, several reports have corroborated that AEP, CtsB, CtsL, and CtsS retain efficient (although suboptimal) activity at neutral pH [40,41,54,153,154].

Together, all these reports demonstrate that lysosomal proteases retain their activity outside the lysosomal compartment at neutral pH, further reinforcing the biological relevance of their non-canonical, extralysosomal activities in the regulation of initially unexpected biological processes.

### 3.2. Extralysosomal Trafficking of Lysosomal Proteases

Another major limitation regarding the non-canonical, extralysosomal activities of these proteases is related to their trafficking. Lysosomal proteases are synthesised as inactive pro-forms that need to be trafficked to the endolysosomal compartment for processing and activation at acidic pH [27]. In this context, lysosomal proteases can reach the extracellular space through lysosomal exocytosis or through alternative trafficking routes [155,156,157], where these proteases have been shown to play key roles, both under physiological and pathological conditions (further discussed in the upcoming sections).

Remarkably, recent reports have started to expand the non-canonical, extralysosomal activities of these proteases through the identification of both cytosolic and nuclear targets, both under physiological and pathological conditions. However, the precise mechanism explaining how lysosomal proteases reach these compartments remains one of the most intriguing questions in the field. Importantly, recent advances have started to unravel how these proteases can reach these extralysosomal compartments. In this regard, cytosolic expression without trafficking through the endoplasmic reticulum has been reported, revealing that some human lysosomal proteases can be expressed as transcript variants lacking the signal peptide (i.e., CtsL) [55,56,158]. However, other human lysosomal proteases (CtsD, CtsS, and CtsV), also linked to non-canonical, extralysosomal activities, only show transcript variants coding for the full-length protein, thus including the signal peptide [54]. Even though, in some specific cases, such as human CtsL, the existence of transcript variants could explain their cytosolic/nuclear localisation, currently there is little evidence to support this as the general mechanism controlling the extralysosomal localisation and activity of these proteases.

Other reports have suggested that the spatially and temporally controlled leakage of lysosomal proteases can, at least during cell division and under physiological conditions, explain the extralysosomal localisation of some of these proteases (i.e., CtsB and CtsL) [21]. This could allow us to understand how lysosomal proteases lacking transcript variants devoid of signal peptide can reach the cytosolic–nuclear compartments of the cell. Moreover, under pathological conditions, such as cancer [159], and under some stress conditions, such as DNA damage [160] or elevated levels of reactive oxygen species linked to high glucose levels in diabetic patients [161], the lysosomal membrane can become leaky, thus potentially allowing lysosomal proteases to reach the cytosolic and nuclear compartments. All these reports indicate that low levels of lysosomal membrane permeability (LMP) are compatible with cell survival, thus raising the possibility of a more generalised way of controlling the cytosolic/nuclear release of lysosomal proteases through the regulation of LMP [17,21,56]. However, the mechanisms regulating the controlled permeabilization of the lysosomal membrane need to be fully characterised.

Finally, extracellular metalloproteases MMP-7, MMP-12, and MMP-14, which are associated to the cellular surface, have been shown to reach the cytosolic/nuclear compartments of the cell through a cell surface-to-cytosol translocation [162,163,164]. As discussed above, lysosomal proteases reach the cell surface and the extracellular space through exocytosis, where they remodel the ECM. Hence, lysosomal proteases, upon extracellular release, could reach the cytosolic/nuclear compartments of the cell in a similar fashion as the cell surface-to-nucleus translocation described for extracellular metalloproteases.

All these reports have started to reveal how these lysosomal proteases escape the lysosomal compartment, thus allowing them to reach extralysosomal locations, where they target specific proteins, controlling initially unexpected biological processes.

### 3.3. Roles Under Physiological Conditions

For a long time, lysosomes have been considered as “suicide bags” filled with acid hydrolases, able to digest the cellular content if they happen to be released in the cytosolic compartment [153], with their role in programmed cell death under physiological conditions recently demonstrated [44] (Figure 2). Therefore, the idea that lysosomal proteases become denatured at neutral pH [38], combined with the elevated content of an ample set of acid hydrolases able to completely digest the whole cellular content, led to the idea that the activity of these lysosomal proteases was restricted to the intraluminal space of lysosomes, thus hampering our understanding of their extralysosomal functions. However, recent reports are revealing non-canonical functions for these proteases in the extracellular milieu, but also in the cytosolic and nuclear compartments of the cell under physiological conditions (Figure 2).

#### 3.3.1. Extracellular Functions

Under physiological conditions, in the extracellular space, several lysosomal proteases (e.g., CtsB, CtsD, CtsK, CtsL, CtsS, CtsX and AEP) play key roles in hormone processing [154], wound healing [165,166,167], bone remodelling [158], membrane repair [168], and in the regulation of cell proliferation, adhesion, and migration [169,170] through the remodelling of the ECM (Figure 2).

In this regard, CtsB has been shown to degrade the ECM, promoting wound healing in normal human epidermal keratinocytes [171]. In other examples, CtsX has been shown to play a key role in the activation of dendritic cells through the processing of β2 integrin receptor Mac-1 [172]. Similarly, CtsX and CtsZ specifically cleave the β2 integrin receptor Leukocyte Function-Associated Antigen-1 (LFA-1), thus promoting the proliferation of T cells and potentially playing a role in T cell morphology [173,174].

For this reason, elevated levels of lysosomal proteases in the extracellular medium have been associated to cancer progression [161,175,176] (further expanded in the “Roles under pathological conditions” section, Section 3.4).

#### 3.3.2. Cytosolic Functions

##### Cytoskeleton

Under physiological conditions, some of these lysosomal proteases have been shown to locate within the cytosolic compartment of the cell, where they target specific proteins, playing unexpected roles in the regulation of the cytoskeleton, thus controlling cell motility and migration. For example, cytosolic CtsL targets dynamin in mouse kidney podocytes, leading to the reorganisation of their cytoskeleton [177]. Furthermore, in the kidney, CtsL in the cytosol of human and mouse podocytes also targets synaptopodin, which plays an essential role in forming intercellular junctions, key to maintaining a normal renal function [178].

##### Cell Death

Several lines of evidence have demonstrated the role that lysosomal proteases play in the activation of different forms of programmed cell death, specifically lysosomal-mediated programmed cell death (LM-PCD) and necroptosis.

In LM-PCD, CtsB, D and L upon release from the lysosomal compartment have been shown to target Bid, a pro-apoptotic member of the Bcl-2 family, leading to a truncated active known as tBid. Upon activation, tBid translocates to the mitochondrial membrane, resulting in cytochome c release, resulting in the activation of caspase, thus initiating apoptosis [43]. Furthermore, lysosomal proteases also facilitate apoptosis through the degradation of antiapoptotic members of the Bcl-2 family, such as Bcl-xL, Mcl-1 and XIAP [43,179]. Additionally, CtsD has been shown to activate apoptosis by directly activating caspase-8 without activation of Bid [180]. Interestingly, during mammary gland involution in a pathway that is activated via oncostatin M/STAT3, lysosomal proteases (i.e., CtsB and CtsL) have been recently shown to trigger cell death independently of the activation of caspases, thus revealing a novel form of regulated programmed cell death [44,181].

Moreover, recent works have revealed a novel role for lysosomal proteases in the activation of necroptosis, a regulated, programmed form of necrosis. In this regard, Liu et al. recently demonstrated that during necroptosis, Mixed-lineage kinase-like protein (MLKL) translocates and polymerizes on the lysosomal surface, resulting in lysosomal membrane permeabilization, leading to the release of lysosomal proteases and the completion of necroptosis [182]. Furthermore, the authors demonstrated that CtsB inhibition completely abolished necroptosis, thus reinforcing a central role for lysosomal proteases in this form of cell death [182]. Furthermore, a key role for both CtsB and CtsS in the regulation of necroptosis through the processing of Receptor-interacting serine/threonine kinase 1 (Rip1) has been reported in macrophages, further reinforcing the role of lysosomal proteases in the regulation of necroptosis [183].

All these reports reveal the complex role these lysosomal proteases play in the activation and regulation of different forms of cell death, thus offering the possibility to exploit their role in cell death for the treatment of cancer.

##### Inflammation

Lysosomal proteases have also been linked in the activation of inflammatory responses through the activation of the inflammasome. In this regard, lysosomal- mediated permeabilization has been shown to drive the activation of the NLRP3 inflammasome. Upon activation, pro-caspase 1 is activated, leading to the activation of interleukin (IL)-1β and interleukin-18, as well as gasdermin D-mediated pyroptotic cell death [184]. Interestingly, genetic ablation of CtsB, C, S, L and Z has been shown to result in reduced IL-1β secretion [185,186,187] and the release of lysosomal proteases has been shown to be required for the activation of the NLRP3-inflammasome [175,188]. Importantly, cathepsins do not directly process and activate caspase 1 or IL-1β, thus suggesting they play a role upstream in the activation of the inflammasome [185].

#### 3.3.3. Nuclear Functions

Lysosomal proteases, through the regulation of specific extralysosomal targets, have been recently linked to immune cell differentiation (Figure 2). Among the different subsets of CD4^+^ T cells, regulatory T cells (Tregs) represent a key subpopulation of T cells in both immune suppression and tolerance. We have recently demonstrated a key role for nuclear AEP in the in vivo regulation of Tregs differentiation through the specific regulation of FoxP3 levels. Indeed, we were able to demonstrate how AEP is able to proteolytically digest FoxP3, and to further demonstrate in vivo how the extralysosomal activity of AEP, through the regulation of FoxP3 stability, regulates the differentiation of Tregs in a mouse model [20].

Similarly, lysosomal proteases have also been linked to transcriptional regulation by directly controlling the levels of specific nuclear targets through their extralysosomal activities (Figure 2). The transcriptional factor CCAAT displacement protein/Homeobox protein cut-like 1 (CDP/Cux) is specifically targeted and digested by nuclear CtsL [176,189], thus resulting in accelerated cell cycle progression and changes in gene expression [13]. Interestingly, nuclear CtsL is also involved in transcriptional regulation through the specific processing of histone 3. In this seminal paper, the authors demonstrated that, during mouse embryonic stem cell differentiation, nuclear CtsL specifically cleaved non-acetylated histone 3 at L21, linked to transcriptionally silent, repressed chromatin regions [17]. More recently, a similar role has been described for both CtsB and CtsL during mouse neural stem cell differentiation [190], thus suggesting that this is a generalised role for lysosomal proteases in the nuclear compartment of cells. Remarkably, a similar role has been described for nuclear CtsD in the processing of histone 3 during mammary gland involution [191].

Another nuclear, non-canonical role for CtsB and CtsL has been recently reported [21]. In this work, the authors demonstrated that both lysosomal proteases play a key role in the nucleus of cells during cell division (Figure 2). Specifically, both lysosomal proteases, through the specific cleavage of histone 3 at residue Y100, contribute to the accurate segregation of chromosomes in mammalian cells. Furthermore, the lack of cleavage of histone 3 during mitotic entry resulted in a significant increase in chromosome segregation defects and accumulation of micronuclei. Therefore, this work demonstrates that, under physiological conditions, both CtsB and CtsL contribute to proper mitosis and maintenance of genomic stability.

### 3.4. Roles Under Pathological Conditions: Cancer

As described in the previous section, non-canonical, extralysosomal functions of these lysosomal proteases have been identified under physiological conditions, demonstrating that these proteases control key biological processes when located outside the lysosomal compartment. However, how the dysregulation of these physiological activities contributes to the onset and progression of pathological conditions, such as cancer, remains vastly unexplored.

In this section, we will summarise our current knowledge about the role of these proteases under pathological conditions, specifically focusing on the onset and progression of cancer.

#### 3.4.1. Role of Lysosomal Proteases in the Extracellular Space in Cancer

Lysosomal proteases, as a heterogeneous group of proteases, are overexpressed in a plethora of human solid tumours, in most cases correlating with poor prognosis and reduced overall survival. Interestingly, several lysosomal proteases have been shown to be released to the extracellular space, where they play key roles in the onset and progression of cancer. In the extracellular milieu, some of these proteases are involved in the remodelling of the ECM, thus playing a key role in cancer cell migration and metastasis [161,175,176], but also in angiogenesis, by allowing the generation of new blood vessels to support tumour growth [53,56,192,193] and promoting the epithelial- to- mesenchymal transition (EMT) [194,195,196,197,198] (Figure 2).

##### Lysosomal Proteases in the Remodelling of the Extracellular Matrix

Several lysosomal proteases have been shown to play a key role in the remodelling of the ECM, thus contributing to the migration, invasion and metastasis of cancer cells. In this regard, CtsB, CtsD, CtsK, CtsL, CtsS, and AEP have been shown to directly participate in ECM remodelling through the proteolytic degradation of collagen, fibronectin, and laminin, among others. Furthermore, these proteases, upon release, can activate other proteases, including other lysosomal proteases or metalloproteases, thus further contributing to ECM remodelling [149,192,193,199,200,201,202,203,204,205,206,207,208]. The identification of the key lysosomal proteases linked to metastasis in specific types of cancer could provide the opportunity to design specific inhibitors against lysosomal proteases with the potential to block cancer metastasis.

##### Lysosomal Proteases in the Epithelial-to-Mesenchymal Transition

EMT is a genetic programme, activated in cancer cells, that allows them to detach from the epithelium, acquiring mesenchymal features and thus playing a key role in cancer progression and metastasis [209]. Lysosomal proteases, besides their contribution to metastasis through the remodelling of the ECM, thus allowing cancer cells to invade new tissues, are directly involved in the EMT. Several lysosomal proteases have been shown to directly regulate the levels of different adhesion proteins, a key event during EMT. For example, CtsB, CtsL, and CtsS have been shown to extracellularly target the extracellular domain of E-cadherin and other adhesion molecules, thus contributing to EMT in cancer cells [40,210]. Similarly, CtsV extracellularly targets E-cadherin [210], but also JAM-2 [211], thus contributing to EMT in cancer cells. Furthermore, the proteolytic degradation of CDP/Cux by CtsL in the nuclei of glioma cancer cells has also been linked to the acquisition of a mesenchymal phenotype [212,213].

##### Lysosomal Proteases in Angiogenesis

Angiogenesis represents a key event during tumour development and progression, as it provides the nutrients and oxygen required to support tumour growth [214]. Beyond their role in promoting angiogenesis through the direct cleavage of ECM components, secreted lysosomal proteases have been shown to promote angiogenesis by other mechanisms. In this context, CtsB promotes angiogenesis by releasing growth factors bound to ECM proteins such as the Vascular Endothelial Growth Factor (VEGF) and Tumour Growth Factor (TGF)-β [215], but also through the direct degradation and inhibition of inhibitors of angiogenesis associated to the ECM, such as TIMP-1 and TIMP-2 [216].

Other lysosomal proteases, such as CtsD and CtsS, act as pro-angiogenic molecules through their extralysosomal activity. CtsD promotes angiogenesis by proteolytically digesting and releasing the Basic Fibroblast Angiogenic Factor (bFGF) and through the direct activation of VEGF [217,218]. On the other hand, CtsS plays a central role in the generation of pro-angiogenic γ2 fragments through the proteolytic processing of laminin in the extracellular milieu, but also promoting the activation of VEGF [206,219].

These studies reveal the noncanonical, extralysosomal activities of lysosomal proteases in promoting tumour progression through the regulation of angiogenesis, offering new opportunities for clinical intervention limiting angiogenesis and cancer cell proliferation by inhibiting their extracellular activity.

#### 3.4.2. Nucleo-Cytosolic Roles of Lysosomal Proteases in Cancer Cells

A wide array of lysosomal proteases are overexpressed in a plethora of human solid tumours, correlating with poor prognosis and reduced overall survival [161,175,176,211]. Interestingly, recent efforts have allowed researchers to unravel some of the cytosolic and nuclear targets of these proteases, thus allowing us to understand their role in the regulation of initially unexpected biological processes and in the onset and progression of cancer (discussed in the next section). Furthermore, recent proteomic efforts from different labs have contributed a wealth of information regarding novel, potential nuclear and cytosolic targets of these lysosomal proteases [40,41,42], expanding even more the number of biological processes potentially regulated by these proteases.

Cancer cells, by using different approaches, have been able to develop strategies to avoid the activation of apoptosis in response to different stimuli [220]. This adaptation provides them with a vast adaptive advantage, enabling them to survive for longer periods and allowing them to accumulate a large number of mutations that can contribute to increased metastatic potential, angiogenesis, cell proliferation, and affect differentiation, without leading to cell death. Interestingly, cancer cells are specially sensitive to lysosomal-mediated programmed cell death (LM-PCD) through the cytosolic activation of pro-apoptotic (i.e., Bid) and the inactivation of anti-apoptotic Bcl-2 family members (Bcl-2, Bcl-XL, and Mcl1) by lysosomal proteases [191,221,222], thus potentially providing novel therapeutical opportunities for cancer treatment [155,179,223,224,225]. Interestingly, upon release into the cytosolic compartment, a wide array of lysosomal proteases (i.e., CtsB, CtsD, CtsL, and CtsG) have been shown to mediate the activation of caspases, both indirectly through the cleavage and inactivation of their inhibitor XIAP, or by directly activating caspases-3, -7, and -8, or through the activation of Bid, thus triggering mitochondrial outer membrane permeabilization, cytochrome c release, and the activation of the intrinsic apoptotic pathway [45,191,194,226,227]. Therefore, the increased sensitivity of cancer cells to LM-PCD offers new clinical opportunities to exploit this weakness to trigger cell death specifically in cancer cells.

Remarkably, a growing number of bona fide targets and functions for some of these lysosomal proteases both in the cytosol and the nuclei of different types of cancer cells have been reported. These specific, emerging new functions of lysosomal proteases offer molecular targets for the design of new strategies for cancer treatment. A summary of the link between some lysosomal protases and specific human tumours, as well as some of the currently identified, biologically significant targets allowing us to understand the extralysosomal role of these proteases in the onset and progression of cancer is presented below.

##### Cathepsin B

**Figure 3:**
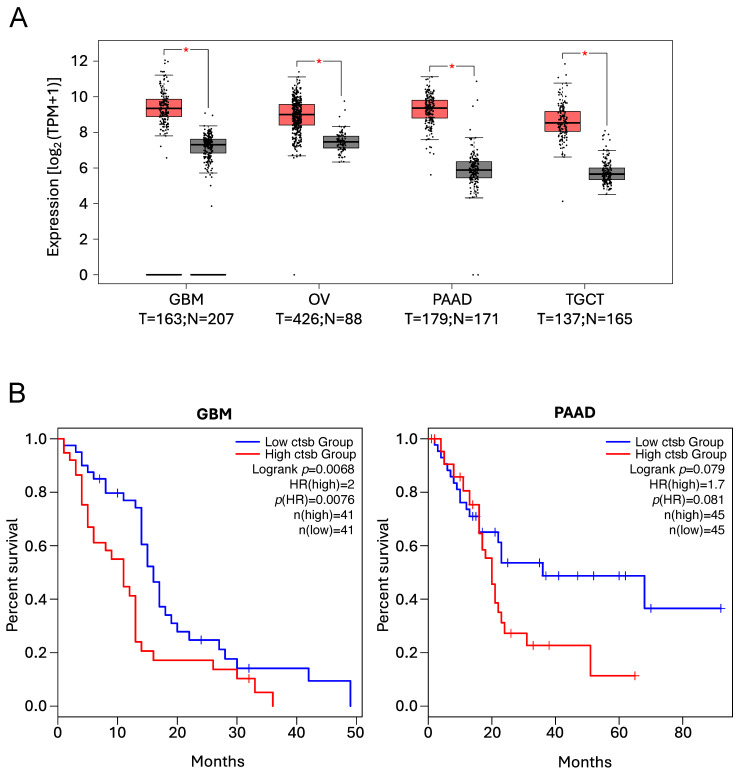
*CTSB* expression levels and Kaplan–Meier analyses illustrating the positive correlation between high *CTSB* expression levels and poor prognosis and reduced overall survival, as previously reported [228,229,230,231,232,233,234,235]. Data were obtained using the GEPIA2 online tool to analyse The Cancer Genome Atlas (TCGA) database. (**A**) Expression levels of *CTSB* in Glioblastoma (GBM), Ovarian serous cystadenocarcinoma (OV), Pancreatic adenocarcinoma (PAAD) and Testicular Germ Cell Tumours (TGCT) patients. (T = tumour, red boxes, N = normal, grey boxes). * *p* < 0.01. (**B**) Kaplan–Meier analyses showing the overall survival in patients expressing low (blue line) vs. high (red line) *CTSB* levels in GBM and PAAD patients.

Differentially expressed gene analyses using The Cancer Genome Atlas (TCGA) database with the GEPIA2 online tool illustrate that CtsB is significantly upregulated in a variety of types of cancer, with its overexpression leading to a worse prognosis and reduced survival in a variety of human solid tumours, as previously reported [228,229,230,231,232,233,234,235] (Figure 3).

Recent works are starting to reveal the role of CtsB in the onset and progression of cancer. In the case of colorectal cancer, CtsB plays a pivotal role in tumorigenesis, cell invasion, and metastasis, through both intra- and extra-lysosomal functions. In this regard, CtsB colocalised with Cyclin-dependent kinase inhibitor 1B (p27^kip1^) within the lysosomal compartment, directly controlling its levels. Therefore, CtsB, through the proteolytic degradation of p27^Kip1^, promotes the accumulation of cyclin B1, thus facilitating cancer cell proliferation. Furthermore, the authors, using a highly selective, non-permeant CtsB inhibitor, demonstrated that the extracellular activity of CtsB is key in promoting cancer cell migration and invasion, thus revealing a non-canonical, extralysosomal activity for CtsB in colorectal cancer metastasis [236].

Interestingly, a recent report has revealed an unexpected role for CtsB in glioblastoma resistance to radiation. The authors demonstrated that CtsB is upregulated in glioblastoma cells in response to radiation, contributing to radioresistance by promoting DNA homologous recombination, although the molecular details are still unknown [237]. Similar functions were recently reported for CtsL, a lysosomal protease with overlapping functions with CtsB [112,221], in human glioma cells. Upon irradiation, CtsL expression levels were significantly increased, resulting in its accumulation within the nuclear compartment of glioma cells. Furthermore, its inhibition sensitised glioma cells to irradiation [222,238]. Interestingly, a recent report has also identified CtsL as a novel player in DNA repair, further validating these observations [239]. Some of the molecular targets regulated by nuclear CtsL have been identified, thus providing the mechanistic insight explaining the role of nuclear CtsL in DNA damage response and radioresistance (see Cathepsin L section for further details). Thus, it would be interesting to check whether CtsB targets the same proteins as CtsL, or whether its role in DNA repair and radioresistance is completely unrelated.

##### Cathepsin D

**Figure 4:**
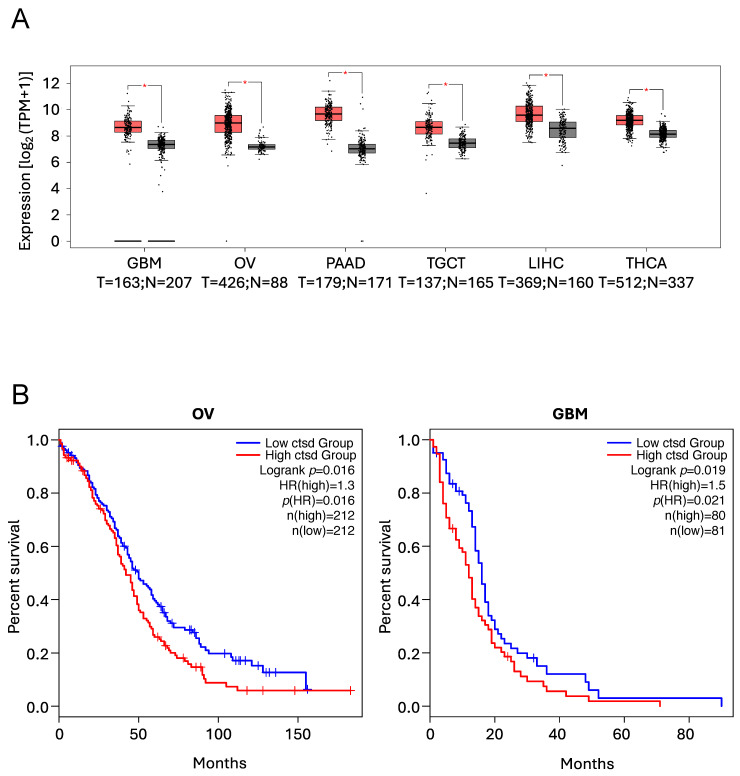
*CTSD* expression levels and Kaplan–Meier analyses illustrating the positive correlation between high *CTSD* expression levels and poor prognosis and reduced overall survival, as previously reported [193,226,227,240,241,242,243,244]. Data were obtained using the GEPIA2 online tool to analyse The Cancer Genome Atlas (TCGA) database. (**A**) Expression levels of *CTSD* in Glioblastoma (GBM), Ovarian serous cystadenocarcinoma (OV), Pancreatic adenocarcinoma (PAAD), Testicular Germ Cell Tumours (TGCT), Liver Hepatocellular Carcinoma (LIHC) and Thyroid Carcinoma (THCA) patients (T = tumour, red boxes, N = normal, grey boxes). * *p* < 0.01. (**B**) Kaplan–Meier analyses showing the overall survival in patients expressing low (blue line) vs. high (red line) *CTSD* levels in OV and GBM patients.

Several reports have demonstrated that CtsD is overexpressed in a variety of human tumours, with this overexpression positively correlating with poor prognosis and reduced overall survival [193,226,227,240,241,242,243,244], as illustrated in Figure 4.

Nuclear CtsD has been reported to specifically target the nuclear repressor Tricho-rhino-phalangeal syndrome 1 (TRPS1) and the nuclear chaperone Scythe/BAG6 (BAT3), controlling cell cycle progression and transformation of breast cancer cells. In this case, the authors demonstrated the nuclear localisation of CtsD, where it was specifically bound to the chromatin fractions, thus reinforcing the nuclear functions of CtsD. Furthermore, the authors went on to demonstrate the nuclear co-localisation of CtsD with both TRPS1 and Scythe/BAG6. Interestingly, in this case, the role of CtsD seemed to be independent of its proteolytic activity, thus raising the possibility that CtsD has nuclear activities unrelated to its traditional enzymatic activity [245].

##### Cathepsin L

**Figure 5:**
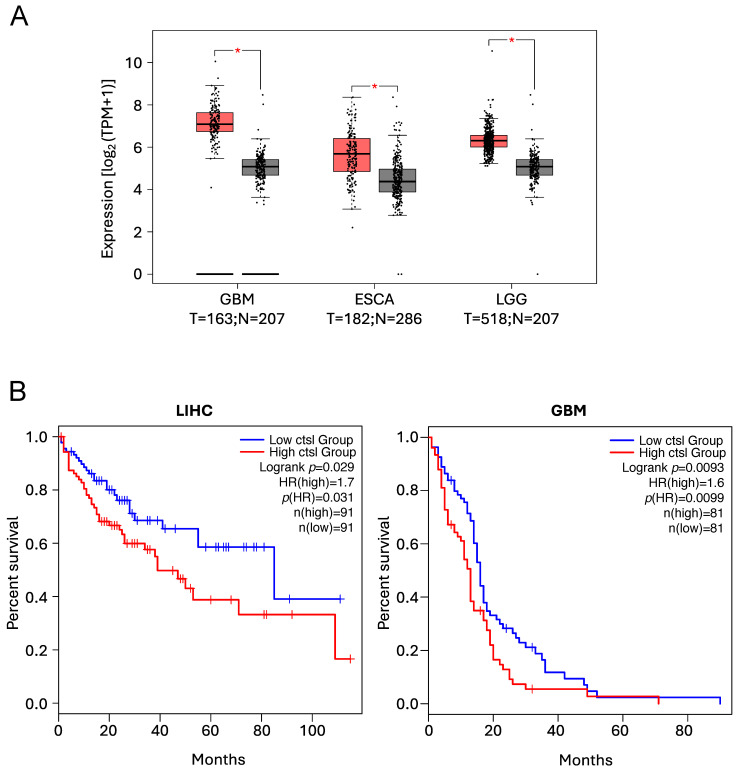
*CTSL* expression levels and Kaplan–Meier analyses illustrating the positive correlation between high *CTSL* expression levels and poor prognosis and reduced overall survival, as previously reported [51,222,238,246,247,248,249,250,251,252,253,254]. Data were obtained using the GEPIA2 online tool to analyse The Cancer Genome Atlas (TCGA) database. (**A**) Expression levels of *CTSL* in Glioblastoma (GBM), Esophageal Carcinoma (ESCA) and Brain Lower Grade Glioma (LGG). (T = tumour, red boxes, N = normal, grey boxes). * *p* < 0.01. (**B**) Kaplan–Meier analyses showing the overall survival in patients expressing low (blue line) vs. high (red line) *CTSL* levels in LIHC and GBM patients.

CtsL has been reported to be overexpressed in a vast majority of human cancers, with its overexpression being a marker for poor prognosis and reduced overall survival [51,222,238,246,247,248,249,250,251,252,253,254], as illustrated in Figure 5.

CtsL is one of the best studied lysosomal proteases regarding its nuclear activity not only in cancer, but also under physiological conditions. As a matter of fact, it is among the first lysosomal proteases described to localise within the nuclear compartment [255]. Interestingly, recent studies have started to identify the specific targets and the biological processes regulated by this protease outside the lysosomal compartment, thus allowing us to rationalise its nucleo-cytosolic localisation.

In 2004, a seminal work in the field revealed an unexpected role for nuclear CtsL by proteolytically processing CDP/Cux, promoting the transition from G1 to S phase, being proposed as a novel mechanism of cell transformation contributing to tumorigenesis [13,176]. Remarkably, in gastric cancer, it has been demonstrated that the proteolytic processing of CDP/Cux by nuclear CtsL induces angiogenesis by altering the gene expression pattern, thus promoting cancer cell survival [256]. In this study, the authors further demonstrated the nuclear role of CtsL, showing that patients with increased nuclear levels of CtsL presented reduced overall survival compared to patients showing low levels of nuclear CtsL.

In 2006, another role for CtsL in the nuclei of mouse embryonic stem cells during differentiation through the regulation of the levels of non-acetylated histone 3 was reported [17], thus revealing a potential role for CtsL in the regulation of gene expression through the removal of epigenetic marks in the N-tail of histones [257]. Remarkably, in colon cancer cells, a role for nuclear CtsL in cell proliferation and cell cycle progression was reported. The authors demonstrated that nuclear CtsL promotes cell cycle progression and proliferation of colon cancer cells by specifically targeting histone 3. In agreement with these observations, colorectal cancer patients expressing high levels of CtsL showed worse prognosis and reduced overall survival when compared to patients expressing low CtsL levels [258]. The authors further confirmed the nuclear role of CtsL, showing, both by Western blotting and confocal microscopy, that it specifically accumulated in the nuclei of colon cancer cells during the G1/G0 phase and that it accumulated in the lysosomal compartment during the S and G2/M phases. Furthermore, the authors were able to demonstrate that nuclear CtsL retained its activity, reaching its maximum at the G1/G0 phase, thus validating its accumulation during this phase of the cell cycle.

In breast cancer cells, it has been reported that the loss of Breast Cancer Type 1 Susceptibility Protein (BRCA1) triggers the nuclear degradation of TP53-binding protein 1 (TP53BP1). TP53BP1 is a double-strand break (DSB) repair protein that, in the absence of BRCA1, serves as a molecular replacement triggering cell cycle arrest and cell death in response to DNA damage. However, its CtsL-mediated degradation allows cancer cells to avoid cell growth arrest and reduce cell death in response to DNA damage. The authors further confirmed the nuclear localisation of CtsL in triple negative breast cancer patients, confirming a negative correlation between the levels of nuclear CtsL and TP53BP1, further reinforcing the nuclear activity of CtsL. Furthermore, this nuclear role of CtsL in breast cancer cells has been shown to play an important role in cancer resistance to treatment [49], thus offering the possibility of designing novel approaches aimed at sensitising cancer cells to conventional chemotherapy and radiotherapy approaches aimed at inducing DNA damage.

Finally, in breast cancer patients, nuclear CtsL has been shown to interact with the Cyclin-Dependent Kinase 2-associated Protein 1 (CDK2AP1), an inhibitor of CDK2, thus revealing a potential role for CtsL as a regulator of cell cycle in breast cancer cells, contributing to aberrant cancer cell proliferation [259].

##### Cathepsin V

**Figure 6:**
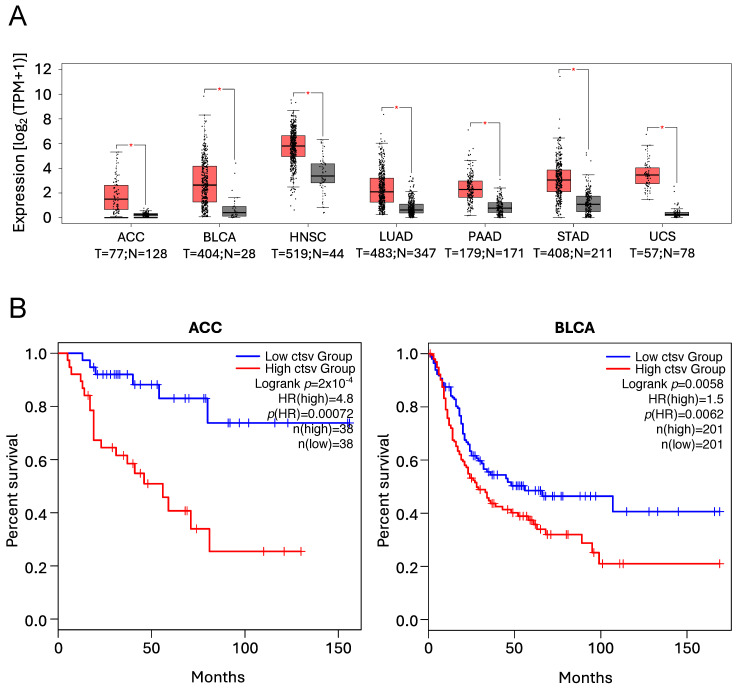
*CTSV* expression levels and Kaplan–Meier analyses illustrating the positive correlation between high *CTSV* expression levels and poor prognosis and reduced overall survival as previously reported [260,261,262,263,264,265,266,267]. Data were obtained using the GEPIA2 online tool to analyse The Cancer Genome Atlas (TCGA) database. (**A**) Expression levels of *CTSV* in Adrenocortical Carcinoma (ACC), Bladder Urothelial Carcinoma (BLCA), Head and Neck Squamous Cell Carcinoma (HNSC), Lung Adenocarcinoma (LUAD), Pancreatic Adenocarcinoma (PAAD), Stomach Adenocarcinoma (STAD), and Uterine Carcinosarcoma (UCS) patients. (T = tumour, red boxes, N = normal, grey boxes). * *p* < 0.01. (**B**) Kaplan–Meier analyses showing the overall survival in patients expressing low (blue line) vs. high (red line) *CTSV* levels in ACC and BLCA patients.

CtsV, also known as CTSL2, a protein highly related to CtsL, has been shown to be overexpressed in a variety of human tumours, with high expression correlating with worse prognosis and reduced overall survival [260,261,262,263,264,265,266,267], as illustrated in Figure 6.

CtsV is not expressed in mouse, thus limiting our understanding of its potential role in the onset and progression of human tumours. However, the nuclear localisation of CtsV has been reported in the case of thyroid carcinoma cells, contributing to the increased proliferation of cancer cells, specifically accumulating in the nuclei of these cells during the S phase [261]. Furthermore, in the case of breast cancer cells, nuclear CtsV has been reported to suppress the expression of the Trans-acting T-cell-specific transcription factor (GATA3) and promoting the stability of both histone 3 and histone 4 through the regulation of the chaperone sNASP [260], thus potentially contributing to cell proliferation and the regulation of gene expression. Furthermore, the authors demonstrated that nuclear CtsV accumulates specifically during the S and G2/M phases, with CtsV knock-down resulting in G2/M arrest.

##### Asparaginyl Endopeptidase

**Figure 7:**
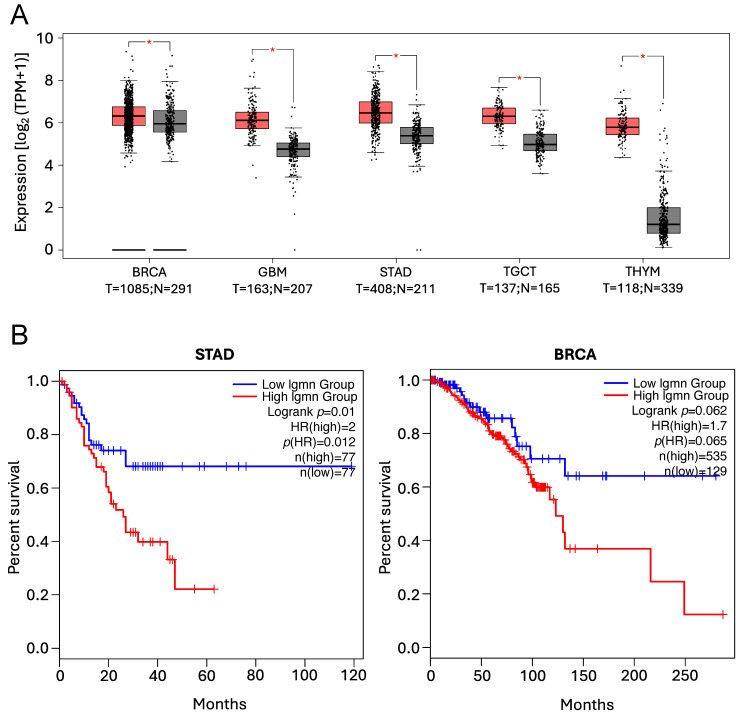
*AEP* expression levels and Kaplan–Meier analyses illustrating the positive correlation between high *AEP* expression levels and poor prognosis and reduced overall survival, as previously reported [48,50,202,268,269,270,271,272,273,274,275,276,277,278]. Data were obtained using the GEPIA2 online tool to analyse The Cancer Genome Atlas (TCGA) database. (**A**) Expression levels of *AEP* in Breast Invasive Carcinoma (BRCA), Glioblastoma (GBM), Stomach Adenocarcinoma (STAD), Testicular Germ Cell Tumours (TGCT) and Thymoma (THYM) patients. (T = tumour, red boxes, N = normal, grey boxes). * *p* < 0.01. (**B**) Kaplan–Meier analyses showing the overall survival in patients expressing low (blue line) vs. high (red line) *AEP* levels in STAD and BRCA patients.

AEP, a unique lysosomal cysteine protease with an exquisite specificity towards asparagine residues in the cleavage sites and evolutionarily related to caspases and separase, is overexpressed in a vast majority of human solid tumours, with its overexpression correlating with reduced overall survival in a variety of human solid tumours [48,50,202,268,269,270,271,272,273,274,275,276,277,278], as illustrated in Figure 7.

In colorectal cancer cells, AEP shows a nuclear localisation, specifically targeting histone 3.1, thus potentially playing a similar role to CtsL in the regulation of gene expression through the regulation of chromatin structure [279]. The authors demonstrated the nuclear localisation of AEP in different colorectal cancer cell lines, confirming that it retains its proteolytic activity at neutral pH and further validating that AEP can efficiently cleave histone 3 at both acidic (pH 5.0) and neutral pH (pH 7.0).

Recently, a novel role for AEP has been identified in glioblastoma. In this study, the authors demonstrated that AEP is overexpressed in tumour-associated macrophages (TAM) in response to hypoxia through HiIF1α regulation. In this context, AEP promotes TAM immunosuppressive polarization via the GSK-3β-STAT3 pathway, thus promoting cancer progression.

Finally, another extralysosomal, non-canonical function for AEP has been recently reported in glioblastoma. In this case, Zhang et al. reported that, under hypoxia and nutrient deprivation conditions, both common features of solid tumours, AEP specifically cleaves DEAD-box helicase 3 X-linked (DDX3X), an ATP-dependent RNA helicase, in the cytosolic compartment of cancer cells. This truncated form of DDX3X translocates and accumulates in the nucleus, triggering alternative RNA splicing that contributes to the adaptation of cancer cells to harsh microenvironments. Thus, this work reveals a novel role for AEP in promoting tumour survival and proliferation through the regulation of nuclear biological processes, such as alternative splicing [280].

## 4. Perspectives

Lysosomal dysfunction is linked to several human maladies affecting millions of people around the world, including LSDs, autoimmune diseases, neurodegenerative disorders, and cancer. Emerging evidence about non-canonical, extralysosomal activities are starting to reveal a more intricate role for lysosomal proteases, both under physiological and pathological conditions, thus providing new clinical opportunities for intervention. Importantly, these extralysosomal activities have been largely overlooked due to the in vitro denaturation of these proteases at neutral pH and their implication in the activation of cell death. However, recent reports have demonstrated that these proteases maintain their activity in the nucleus and cytosol of the cells, where they control key aspects of cell physiology both under physiological and pathological conditions. Therefore, in order to understand the extent of their role both under physiological and pathological conditions, a number of questions still need to be addressed.

In this context, the identification and characterisation of secretory lysosomes have allowed us to understand how these proteases locate to the extracellular compartment. In this extracellular milieu, lysosomal proteases are nowadays known to play key roles in ECM remodelling, angiogenesis, invasion, metastasis, etc. However, although recent advances are starting to shed light on the mechanism that allows these proteases to reach the nucleo-cytosolic compartment of cells, at least under specific conditions, further investigation on this topic is still required. On the other hand, the identification and characterisation of nuclear and cytosolic targets of some of these proteases, both under physiological and pathological conditions, has started to reveal their non-canonical, extralysosomal roles. However, recent proteomics studies have identified novel, potential extralysosomal targets of these proteases. Therefore, a complete characterisation of the proteins targeted by these proteases in the nuclear and cytosolic compartments of the cell and the biological processes they regulate will provide us with a deeper understanding of their roles, but also with novel targets with the potential to be translated into the clinical setting for cancer treatment.

In a pathological context, the canonical role of lysosomes in diseases such as LSDs and autoimmune diseases is well characterised, as described above. However, the accumulating evidence unveiling a non-canonical, extralysosomal activity for these proteases might require considering the possible contribution of these new activities to the onset and development of these diseases. For example, aberrant processing of surface markers by extracellular lysosomal proteases can lead to the generation of new epitopes that could potentially trigger abnormal immune responses, further contributing to the onset and progression of autoimmune diseases.

Importantly, unexpected extralysosomal targets and functions for these lysosomal proteases occurring in the extracellular milieu, but also in the cytosolic and nuclear compartments, where they partake a key role in the onset and progression of cancer, are starting to emerge. This raises two exciting questions: Are these non-canonical, extralysosomal functions exclusively happening under pathological conditions, or are they still unidentified, exacerbated physiological functions of these proteases outside the lysosome, resulting in the onset and progression of these pathologies? Which other processes regulated by these proteases are we still missing, both under physiological and pathological conditions?

Accumulating evidence supports an unanticipated role for lysosomal proteases in the cytosol and nucleus of cancer cells. In this context, these proteases, by targeting specific cytosolic or nuclear targets, contribute to the aberrant cell cycle of cancer cells by targeting key regulators of the cell cycle. Furthermore, lysosomal proteases, through non-canonical, extralysosomal functions, have also been shown to target key proteins involved in the regulation of gene expression, thus contributing to the altered gene expression patterns observed in cancer cells. Moreover, key proteins involved in DNA damage response have been characterised as novel targets of these lysosomal proteases, thus explaining their role in the increased resistance of cancer cells to conventional chemo- and radiotherapy approaches aimed at inducing DNA damage. The characterisation of these molecular mechanisms and the identification of novel targets and biological processes controlled by these proteases has the potential to provide new opportunities for the design of innovative clinical therapies in the treatment of cancer focused on disrupting the role of these proteases in promoting cancer onset and progression.

## Figures and Tables

**Table 1:** Some examples of lysosomal storage diseases indicating the lysosomal gene mutated. Diseases in which mutations in lysosomal proteases have been identified are highlighted in bold.

Group	Disease	Gene
Glycogen storage disease	Pompe disease	*GAA*
	Danon disease	*LAMP2*
Lipidoses	**Niemann-Pick disease type C**	*NPC1*, *NPC2*, ***CTSB***, ***CTSL***
	**Neuronal ceroid lipofuscinoses**	*PPT1*, *TRP1*, ***CTSD***, ***CTSF***, ***CTSB***, ***CTSL***
Lysosomal transport disease	Cystinosis	*CTNS*
	**Pycnodysostosis**	***CTSK***
Mucolipidosis	Type I	*NEU1*
	Type II	*GNPTAB*
Mucopolysaccharidoses	Type I (Hurler syndrome)	*IDUA*
	Type II (Hunter syndrome)	*IDS*
	Type III (Sanfilippo syndrome)	*SGSH*, *NAGLU*, *HGSNAT*, *GNS*
Glycoproteinoses	**Galactosialidosis**	***CTSA***
Sphingolipidosis	Niemann-Pick disease	*SMPD1*
	Fabry disease	*GLA*
	Schindler disease	*NAGA*
	Tay-Sachs	*HEXA*
	Gaucher disease	*GBA*

## Data Availability

Not applicable.
